# A Grid-based solution for management and analysis of microarrays in distributed experiments

**DOI:** 10.1186/1471-2105-8-S1-S7

**Published:** 2007-03-08

**Authors:** Ivan Porro, Livia Torterolo, Luca Corradi, Marco Fato, Adam Papadimitropoulos, Silvia Scaglione, Andrea Schenone, Federica Viti

**Affiliations:** 1 Computer Science, Systems, and Communication Department, University of Genova, Viale Causa 12, 16100 Genova, Italy

## Abstract

Several systems have been presented in the last years in order to manage the complexity of large microarray experiments. Although good results have been achieved, most systems tend to lack in one or more fields. A Grid based approach may provide a shared, standardized and reliable solution for storage and analysis of biological data, in order to maximize the results of experimental efforts. A Grid framework has been therefore adopted due to the necessity of remotely accessing large amounts of distributed data as well as to scale computational performances for terabyte datasets. Two different biological studies have been planned in order to highlight the benefits that can emerge from our Grid based platform. The described environment relies on storage services and computational services provided by the gLite Grid middleware. The Grid environment is also able to exploit the added value of metadata in order to let users better classify and search experiments. A state-of-art Grid portal has been implemented in order to hide the complexity of framework from end users and to make them able to easily access available services and data. The functional architecture of the portal is described. As a first test of the system performances, a gene expression analysis has been performed on a dataset of Affymetrix GeneChip^® ^Rat Expression Array RAE230A, from the ArrayExpress database. The sequence of analysis includes three steps: (i) group opening and image set uploading, (ii) normalization, and (iii) model based gene expression (based on PM/MM difference model). Two different Linux versions (sequential and parallel) of the dChip software have been developed to implement the analysis and have been tested on a cluster. From results, it emerges that the parallelization of the analysis process and the execution of parallel jobs on distributed computational resources actually improve the performances. Moreover, the Grid environment have been tested both against the possibility of uploading and accessing distributed datasets through the Grid middleware and against its ability in managing the execution of jobs on distributed computational resources. Results from the Grid test will be discussed in a further paper.

## Background

It is well known that genomics and proteomics experiments are associated with various and complex laboratory protocols that need to be described in great detail, regarding substances, procedures and conditions used. The large amount of data generated by these experiments requires extensive computational efforts to be interpreted and to produce accurate and biologically consistent predictions. For these reasons, exploitation of gene expression data is fully dependent on the availability and sharing of (i) large sets of genomic and proteomic data and (ii) advanced statistical analysis tools, both being typically collected by distributed databases and providers, and structured under different standards [1]. Therefore, integrative web based services packages are often used in order to provide biologists and bioinformatics with a set of algorithms and services covering the whole range of steps in microarray data analysis. As measurements from experiments can dramatically range in their accuracy and reproducibility, with increasing emphasis on modelling error information, researchers are used to design experiments with more biological replicates [2]. Statistical processing systems can overcome this problem by widening the amount of data they are able to consider, but very large datasets are required to achieve satisfactory results [3,4]. However, cost is a strong limit on the size of experiments. As a solution, similar data may be collected across several acquisition facilities, but side conditions associated to experiments must be tracked in order to be able to reproduce or compare different experimental setups [5]. Furthermore, end-users may be provided with different analysis algorithms by different providers, and search tools may be needed to find data and applications. Eventually, data or experimental setups as well as results from experiments may be collected through a user-friendly web interface. To complete this approach, modular and independent applications may be published on a portal, and either single algorithms or a combination of them may be invoked remotely by users, through a workflow strategy [6].

Due to these reasons, several systems have been presented in the last years in order to manage the complexity of big microarray experiments. Although good results have been achieved, each system tends to lack in one or more fields. The MARS system [5] may keep track of information collected during microarray experiments, with special emphasis on quality management and process control. Several applications for storing, retrieving, and analysing microarray data may be connected through a web service infrastructure. Thanks to the use of ontologies and to standard definitions, studies performed by MARS may be exported to public repositories; however neither data nor applications can be accessed remotely, regardless of SOAP used as internal protocol. XPS [7] is a distributed system for microarray storage and analysis. XPS is intended as a data warehouse and data mining system in the terabyte realm and, based on the ROOT framework [8], it is supposed to run in a Grid context. However, ROOT is an intrinsic part of the system and XPS fully depends on the availability of ROOT all around the Grid infrastructure. The ODD/Genes system [9] provides some basic microarray analysis tools together with a knowledge discovery module and it is mostly based on Sun Microsystems Grid components. It has also the ability to manage workflows for microarray data and to distribute jobs on different resources, but it heavily depends on Sun Technology framework and it has not been tested on a real Grid infrastructure.

## Results

### Grid architecture

A Grid based approach may provide a shared, standardized and reliable solution for storage and analysis of the afore-mentioned biological data, in order to maximize the results of experimental efforts. Services may be implemented within an existing Grid computing infrastructure to solve problems concerning both large datasets storage (data intensive problem) and large computational time (computing intensive problem). A Grid framework has been adopted due to the necessity of remotely accessing large amounts of distributed data. Moreover, although clusters may be computationally more efficient for small data sets, they are hardly able to scale performances in terabytes scenarios.

**Figure 1:**
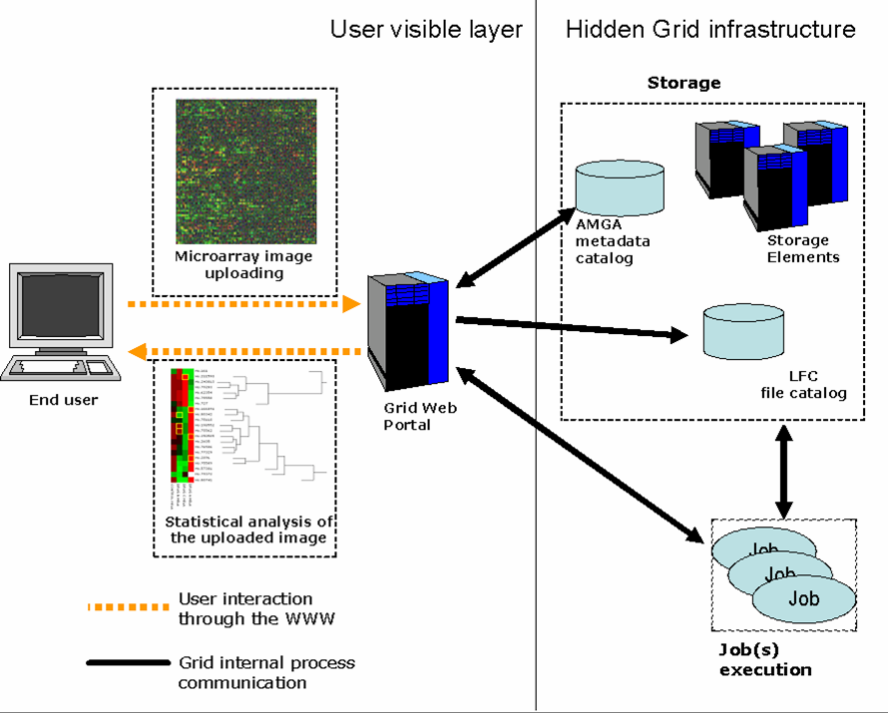
The proposed architecture. On the left, components visible to users, information flow and data are shown. On the right, the complexity of the Grid infrastructure (security framework omitted) is totally abstracted by the Grid portal.

As further element of the architecture, a Grid portal may allow unpractised users to store their experimental data on a complex storage system and to access distributed data and services. Eventually, security and privacy issues can be addressed using a certificate-based authentication schema coming out for free from the Grid technology. Handling biomedical data is a challenging task because users have (i) to store data on secure and reliable repositories and (ii) to describe their data in order to be able to retrieve them whenever requested. Instead of developing a database, we decided to implement a Grid-based data storage and management system. The functional structure of the environment is described in Figure 1. Functions provided by the Grid infrastructure are hidden from users interacting through the portal to submit or search for data (images, parameters, metadata) and applications.

A state-of-art Grid portal is being implementing in order to hide the complexity of framework from end users and to make them able to easily access available services and data. Usability had been kept in mind in designing and implementing the interface, and a really common model has been adopted, which presents a menu with available tools and services on the left and main contents in the middle of the page. This way, the Grid portal, from the user perspective view, is not different from the most used Web-based applications.

**Figure 2:**
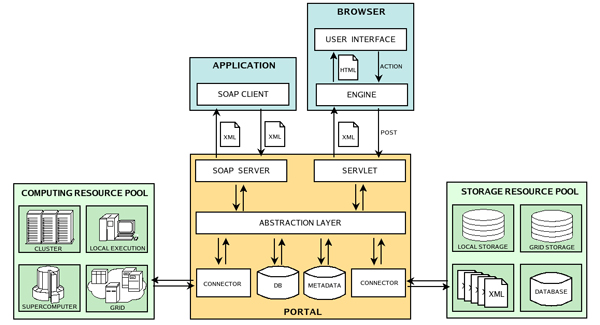
Architectural design of the portal.

The portal (Figure 2) consists in an HTTP server that provides contents by means of queries to metadata catalogues and as a result of data processing. Through the portal users communicate with the available data and computation resources. The following functions may be provided to the users:

• upload of data files with related permissions and metadata

• search of data files through queries on metadata

• list of available algorithms (both local and remote)

• creation of a virtual experiment by associating data and algorithms

• interactive definition of parameters

• asynchronous running of experiments

• control on the status of experiments

• visualization of results

• download of results

With the aid of the portal, administrators can also manage and upgrade information concerning data, algorithms and users.

The portal itself is a Web application written in Java, exposing Java servlets and interacting, through API, with system commands or with services. Interfaces to users may be represented either as a traditional GUI, exposing all available services, or as WSDL pages exposing only some Web Services. Relative to the inputs, the portal has been planned to transform the HTTP requests submitted through the portlets on the interface into XML messages to the server components. Regarding the outputs, the server components, after accessing resources, return XML messages to the portlets. Such messages are then translated in HTML and updated pages are visualized according to the AJAX paradigm [12].

### A general use case

As an example, a general use case scenario is described as follows. The user logs into the portal with his/her username, password and a PIN code. Then he/she can access two different kinds of services: data management and processing. Through the data management tool, user data can be uploaded to the application (actually they are stored into a Grid storage element) and some mandatory description fields are filled in (data to be stored into the metadata catalog). From now on, data are accessible through a search tool. Through this tool, users can then select distributed data, corresponding to their search criteria, coming from several experiments done at different laboratories. Such data can then be processed, as a unique distributed experiment, both locally and on the Grid, by using computation services (custom as well as made available by third parts) deployed through the portal.

As regards microarray experiments, a number of analysis tools are already available. The sequence of these tools just provides a first validation pipeline for our framework but also experiments from different fields of data analysis (medical imaging, signal processing, etc.) may be considered. For the moment, forthcoming microarrays processing tools include (i) group opening and image set uploading, (ii) normalization, and (iii) model based gene expression (based on PM/MM difference model). They are all based on dChip software [13]. A further tool providing the definition of differentially expressed genes is planned but is not available yet. Other tools, typical of the bioinformatics community, like a workflow management system and tools to query and use online resources (data bank, web services) are planned together with the integration and availability of R/Bioconductor based tools [14].

According to our approach, available applications are provided as grid services exploiting the standard Grid infrastructure (authentication, inter-process communication, data management, job scheduling). To complete this Grid approach, monitoring and account services are planned for analyzing usage and performance on the Grid itself.

### Real world scenarios

Without losing generalization, two different scenarios have been considered in order to highlight the benefits that can emerge from our Grid based platform. Firstly, the mRNA expression profiles from the human umbilical vein endothelial cells (HUVEC) treated with three different types of interferons (IFNs) are under study. IFNs are highly pleiotropic cytokines displaying marked angiogenic activity and thus a promising therapeutic activity in many tumor models. This remarkable anti-tumor activity has been related to various mechanisms such as direct inhibition of tumor growth and induction of differentiation and apoptosis, stimulation of specific cellular immune response and inhibition of angiogenesis.

To date the gene expression profile induced by IFNs has been studied mainly in tumor cell lines and some primary cells [15], and little is known about the effects induced by IFNs in endothelial cells compared to other cell types. The definition of cell-type specific profiles emerges therefore as an issue of paramount importance for a fully understanding of the biology of IFNs and their anti-angiogenic activity. To reveal this, 9 total experiments have been performed and gene profiles will be analyzed through our platform including HUVEC cells either from controls cells or from cells treated with recombinant human IFN-a, IFN-b and IFN-c. All cells were processed by using Affymetrix GeneChip HG-U133A microarrays.

The second scenario deals with a different cell-type, the adult human bone marrow derived Stem Cells (SCs). SCs are defined as self-renewal, multipotent progenitor cells with the capacity to differentiate into several distinct cell lineages. In adults of multiple vertebrate species, the bone marrow represents the natural source of Mesenchymal Stem Cells (MSC) and of Haemophoietic Stem Cells (HMS). The properties of MSCs, such as clonogenicity, phenotype and activities, are deeply influenced by the microenvironment cues they are subjected to. Our tissue engineering group is currently performing various experiments including a three-dimensional (3D) perfusion bioreactor system and the typical monolayer (2D) for cell expansion, in order to better prepare progenitors cells to their in vivo tissue generation and repair. It has been shown [16,17] that a 3D micro-environment could provide a significant increase in MSC clonogenicity maintenance, a cell-matrix interaction enhancing, an in-vitro osteogenic differentiation enhancing, and a higher in vivo bone forming efficiency. To further investigate this features, genes profiles of the human bone marrow MSCs expanded either in 3D structures or in 2D ones will be analyzed through our platform by using the Affymetrix GeneChip HG-U133A microarrays, to try to identify genes responsible of the clonogenicity maintenance.

### Performance tests

A gene expression analysis concerning 15928 genes has been performed on a dataset with 258 arrays of Affymetrix GeneChip^® ^Rat Expression Array RAE230A, from the ArrayExpress database. The sequence of analysis includes three steps: (i) group opening and image set uploading, (ii) normalization, and (iii) model based gene expression (based on PM/MM difference model).

First of all, in order to be able to exploit distributed computational resources, two different Linux versions of dChip have been developed. The first one sequentially reproduces the procedure as described above, while the second one, a parallel version of the former one, has been designed and implemented in order to improve the overall execution time. This has been achieved by splitting the code into different parallel modules as follows.

The first module carries out the uploading of the microarray images and their normalization based on invariant set method, while the second one performs the PM/MM gene expression extraction. A further module running the definition of differentially expressed genes has not been implemented yet. The first module had been parallelized against "array": each normalization job take into account only the base array and one or more array from the dataset. We can launch at most N-l normalization jobs where N is the size of the dataset. The second module needs to work on every normalized array because of its intrinsic statistical nature. Starting from this point of view, it has been parallelized against "genes". This means that each job takes M (few hundreds to few thousands) genes from each array and K processing jobs can be run (where M is NumberOfGenesInEachArray/K). The implemented parallelization is a coarse grained one. Data have been distributed on different jobs but algorithms have not been modified because they have been already validated by the research community and therefore they do not have to be handed.

To test the feasibility of this parallel implementation, a comparison has been carried out between the two versions. The serial version has been executed on a Xeon 2.4 GHz processor and execution times were: 22'40" for step (i) + (ii), and 14'19" for steps (iii). These times are measured with dChip internal timings on algorithm steps. As regards the parallel version, the same three steps of the analysis sequence have been executed on a local cluster with 7 different machines (1 Xeon 2.0 GHz, 4 Xeon 2.4 GHz, 1 Pentium4 3 GHz, 1 Athlon 3000). Execution times were: 2'14" for step (i) + (ii), 3'16" for steps (iii). Also these times are measured with dChip internal timings on algorithm steps.

The overall execution times resulted: 36'59" for the serial implementation, and 5'30" for the parallel implementation on the cluster. Therefore, being T(l) the execution time on a single processor, and T(N) the execution time on N different processors (in our case N = 7), the speedup ratio S(N) = T(l)/T(N) is 2219 sec/330 sec = 6.72, that is very close to the ideal one.

From these results, it emerges that actually the parallelization of the analysis process and the execution of parallel jobs on distributed computational resources improve the performances. It is worth noting that these results have been obtained through the parallelization of the code "as is", without refining the dChip implementation. This means that MPI (Message Passing Interface) has been used only to parallelize the running of jobs on different machines without porting the whole code to MPI. Improvements in execution times and robustness are then expected from the full porting of code to MPI in order to exploit its flexibility in automatically managing job allocation among different worker nodes and job communication.

As a final test, the Grid environment is being tested both against the possibility of uploading and accessing distributed datasets through the Grid middleware and against its ability in managing the execution of jobs on distributed computational resources.

The same analysis on the same dataset described above is still in progress on the Grid environment. Tests are being carried out both on the BIOMED infrastructure [18] and on the GILD A test-bed [19]. The up-to-now results of these tests are not sound enough and further improvements are required. For this reason it is decided not to publish them in this paper. A further paper is planned in order to describe the statistical comparison among implementations on different clusters and on different Grid infrastructures with special attention paid to the scaling of performances according to the dimensions of datasets.

However, a number of general considerations may be stated as references for this ongoing comparison:

- the parallel implementation on the cluster is likely to be more efficient for very small experiments

- Grid job submission times due to queues on overcrowded infrastructures may influence execution times. However, job submission time can be considered as a constant within the overall execution time, and therefore it may become less influential as the size of the dataset increases

- although clusters seem to provide better performances for small datasets, they are hardly able to tackle the computational challenges of huge bioinformatics problems. As an example, the first module of our application can be parallelized up to N-l jobs where N is the number of input arrays (usually several thousands). A Grid environment may provide enough CPUs to carry out this scaling but a cluster easily may not.

- data from bioinformatics experiments, as well as services for analysis, are usually located on geographically distributed resources. The Grid framework has been adopted mainly due to its ability to remotely access this large amount of distributed data and resources. A cluster approach is intrinsically unable to provide such functionalities.

## Conclusion

Distributed microarray experimental data and applications may be uploaded and/or searched through the Grid environment described in the paper. Users have only to fill in data describing experiments and then they may use the environment to access microarray bioinformatics services for data management and data storage or to access processing facilities on the Grid. A number of analysis functions are provided: users can run basic processing on data, have a look at intermediate results or come directly to more general conclusions by looking at gene expression levels. Moreover, analysis can be performed just on data provided by the user or also on data coming from other researchers, thus widening the size of available datasets and increasing the possibility of obtaining statistically significant results. On the other hand, distributed computing resources may be used to dramatically reduce computational times for complex experiments. Eventually, bioinformatics data suppliers are provided with a secure and reliable repository for their data and applications as well as researchers are provided with a collection of data and tools for unsupervised data processing, through a simple web based portal interface. Very basic bioinformatics skills are required to access the environment through the portal, allowing also clinical and industrial users to hire services on demand for both data storage and processing. Through our Grid platform, a gene expression analysis based on microarray experiments is planned for the two real world scenarios described above.

Performance tests have been carried out on a dataset with 258 arrays of Affymetrix GeneChip^® ^Rat Expression Array RAE230A, from the ArrayExpress database. Three steps of the analysis sequence have been taken into account: (i) uploading of the image data set, (ii) normalization, (iii) model based gene expression.

For these steps, tests produced very promising results for job parallelization on a small cluster, with a speedup ratio of 6.72. Further improvements are expected from the full porting of code to MPI and from tests on the fourth step of analysis sequence.

As regards the ongoing tests for the Grid implementation, some considerations are needed. In bioinformatics, real world scenarios, data and services have to be collected from geographically distributed resources. Only a Grid framework may remotely access these resources in an efficient and safe way, whereas a cluster approach may not. Although Grid execution times are influenced by the job submission queues on infrastructures, these submission times do not change with the increase in the size of datasets and therefore their relative contribution to overall execution times should decrease for large amounts of data. More efficient and powerful Grid infrastructures may greatly reduce the overload of these submission times. On the other hand, clusters may hardly provide the computational resources (thousands of CPUs) needed in large bioinformatics experiments.

Starting from these considerations, only a Grid approach seems to be suitable for the bioinformatics experiments we are dealing with.

## Methods

The described environment relies on storage services (with replication and catalog services) and computational services provided by the gLite Grid middleware [10]. gLite middleware has been selected for the architecture as the official Grid middleware for the Italian Grid infrastructure and for the EC funded EGEE (Enabling Grids for E-sciencE) project. gLite is composed of a Workload Management System (WMS), a Data Management System (DMS), an Information System (IS) and various monitoring and installation services. Services of the WMS are responsible for the acceptance of submitted jobs and for sending them to the appropriate Computing Elements (depending on job requirements and available resources). The Computing Element (CE) is the Grid interface to a computing cluster and it is defined as a Grid batch queue. The CPUs to be allocated are automatically selected by the Resource Broker (RB) which is the machine where the WMS services run. The User Interface (UI) is the component that allows users to access functionalities of the WMS. Concerning the data management, the DMS relies on two kinds of services: the LCG File Catalog (LFC) and the Storage Element (SE). LFC provides a global hierarchical namespace of Logical File Names (LFNs), which are mapped to physical file names usually stored on a SE. Data are stored on distributed SEs through a script which automatically loads data. At the present time, single copies of data are saved, but several replicas could be done to improve performances. Actually, the Grid environment is also able to exploit the added value of metadata in order to let users better classify and search experiments. A very flexible use of metadata is provided by associating them with LFNs, through the AMGA metadata server: every user can have his/her own metadata associated with a file or have different metadata associated with a file depending on the context. Collected metadata are associated to files stored on Grid through a reference on the LFC catalog system. This way, user information with a special format (VOMS credential) can be mapped to AMGA roles and users can access metadata collections. To this goal, a full security x.509 certificates based framework [11], with Access Control Lists on data and processes, has been deployed in order to guarantee security and auditing. Eventually, through the IS, CEs suitable to run jobs can be located, and SEs holding replicas of Grid files together with the catalogs that keep information of these files can be found. Therefore data are not sent with jobs but they are retrieved from SEs through logical names by using GFAL API.

## Authors' contributions

LC participated in the development of the code, MF participated in the design of the system and helped to draft the manuscript, AP participated in the design of stem cells experiments and carried out the design of microarrays studies, IP participated in the design of the system and carried out the development of the code, SS carried out the design of stem cells and microarrays experiments and helped to draft the manuscript, AS participated in the design of the system and helped to draft the manuscript, LT participated in the design of the system and carried out the development of the code, FV participated in the design of microarrays studies and participated in the design of the system. All authors read and approved the final manuscript.
